# Fusion Based on Visible Light Positioning and Inertial Navigation Using Extended Kalman Filters

**DOI:** 10.3390/s17051093

**Published:** 2017-05-11

**Authors:** Zhitian Li, Lihui Feng, Aiying Yang

**Affiliations:** School of Optoelectronics, Beijing Institute of Technology, 5 S. Zhongguancun Street, Beijing 100081, China; leezhitian@163.com

**Keywords:** indoor positioning, visible light fusion positioning, Kalman filter

## Abstract

With the rapid development of smart technology, the need for location-based services (LBS) increases every day. Since classical positioning technology such as GPS cannot satisfy the needs of indoor positioning, new indoor positioning technologies, such as Bluetooth, Wi-Fi, and Visible light communication (VLC), have already cut a figure. VLC positioning has been proposed because it has higher accuracy, costs less, and is easier to accomplish in comparison to the other indoor positioning technologies. However, the practicality of VLC positioning is limited since it is easily affected by multipath effects and the layout of LEDs. Thus, we propose a fusion positioning system based on extended Kalman filters, which can fuse the VLC position and the inertial navigation data. The accuracy of the fusion positioning system is in centimeters, which is better compared to the VLC-based positioning or inertial navigation alone. Furthermore, the fusion positioning system has high accuracy, saves energy, costs little, and is easy to install, making it a promising candidate for future indoor positioning applications.

## 1. Introduction

The need for a navigation system for both indoor and outdoor settings is increasing every day. For the outdoor case, the Global Positioning System (GPS) is well established and has been widely used. For some outdoor scenes without available satellite signals, inertial navigation can be used as aided technology.

Demand for indoor positioning is increasing because of the increase in the use of smart devices. Many indoor positioning technologies are being proposed and used, such as Wi-Fi, Bluetooth, RFID, and inertia navigation [1,2,3,4]. Besides these, a promising indoor positioning system based on visible light was proposed with visible light communications (VLC) technology recently. It is a promising indoor positioning technology that has the following advantages [5]: firstly, LED light sources are widely used as energy-saving light sources, so VLC positioning services can be provided universally so long as lighting infrastructures exist, which means that the hardware cost is minimized; secondly, VLC positioning can obtain a high position accuracy since the multipath effects will be lower than traditional wireless radio-wave approaches; and third, radio frequency (RF) radiation is hazardous or even forbidden in some places such as hospitals and airplanes, while VLC-based approaches can fit in perfectly, as no RF interference will be generated by LEDs. That is why we regard VLC positioning as a “green positioning method”. However, two main shortcomings of the VLC-based positioning—positioning failure due to the obstruction of visible light and accuracy degradation due to the multipath reflection effect [6]—should be overcome before it is applied.

Inertial navigation is an important positioning technique because it works automatically, and has the benefit of high short-term accuracy and great anti-interference ability. However, inertial navigation has a drawback that it cannot provide long-term accurate positioning because of the cumulative error with time. The authors of [7] verified that the fusing method with visible light positioning will be helpful to achieve a significant reduction in localization errors. In this paper, a fusion positioning method based on an extended Kalman filter is used to fuse the inertial navigation data and visible light positioning data in order to solve the system failure and the problem of decreased accuracy. The performance of the fusion positioning method we proposed demonstrates that it will overcome the shortcomings of visible light positioning and will not accumulate the error of the inertial navigation at the same time. The remainder of this paper is arranged as follows: Section 2 discusses the system configuration of the proposed system and algorithm. The results are presented in Section 3 followed by a related discussion. Finally, conclusions are addressed in Section 4.

## 2. System Configuration and Algorithm

### 2.1. System Design

**Figure 1:**
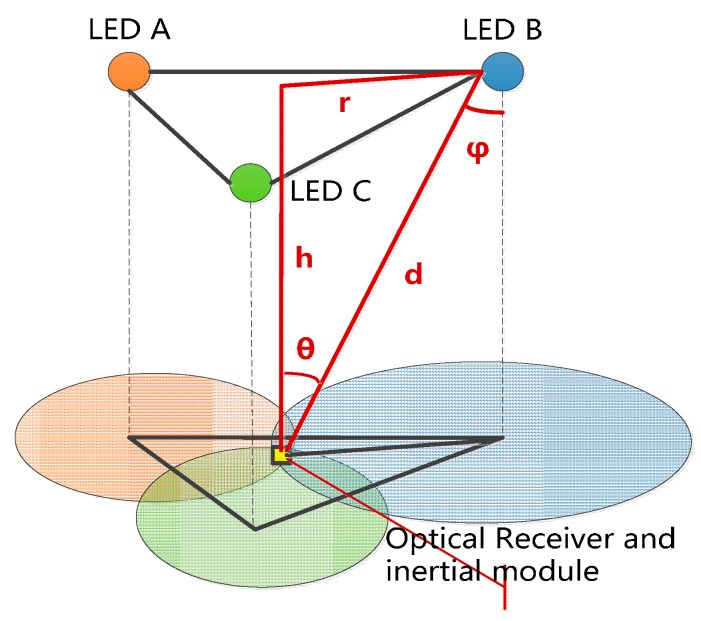
The model of visible light positioning.

Figure 1 depicts the proposed system configuration model for a typical indoor environment. There are several VLC positioning units located on the ceiling, every unit has three LED bulbs and each of these bulbs acts as a single optical transmitter. Every bulb has a unique code assigned to itself, the bulbs can be distinguished through their unique code. The LEDs are modulated in an on-off keying (OOK) format. The receiver, including an optical detector and inertial module, is assumed to be a wearable mobile device.

### 2.2. VLC Positioning Algorithm

The VLC-based positioning data, i.e., the walker indoor coarse position, is acquired in an LED lighting environment with a commonly used trilateral RSS algorithm [8,9]. Based on the Lambert model, the channel gain of the LED bulb can be expressed as Equation (1) [10]:

(1) $$H(0)=\frac{\left(m+1){\mathrm{Acos}}^{m}(\phi )\cos(\theta \right)}{2\pi d^{2}}$$

where A is the physical area of the photodiode detector in the VLC positioning module, θ is the angle of incidence with respect to the receiver axis, ϕ is the angle of incidence with respect to the LED bulb. *m* represents the order of Lambertian emission, and is denoted as

(2) $$m=\frac{-\ln2}{\ln(\cos\Phi_{1/2})}$$

where $\Phi_{1/2}$ is the half power angle of the LED bulb. Generally, ϕ=θ, m=1, cosϕ=h/d, if we denote the light intensity of the LED bulbs transmitter and positioning module receiver as $I_{t}$ and $I_{r}$ respectively, we will achieve Equation (3) as follows:

(3) $$I_{r}=I_{t}\times H(0)=\frac{A}{\pi }\times \frac{I_{t}h^{2}}{d^{4}}=C\times \frac{I_{t}h^{2}}{d^{4}}$$

As shown in Figure 1, we obtain Equations (4) and (5) based on Equation (3), which demonstrates the relationship among r, d, and h as follows:

(4) $$r=\sqrt{d^{2}-h^{2}}={\left(\sqrt{C\times h^{2}\times I_{t}/I_{r}}-h^{2}\right)}^{\frac{1}{2}}$$

(5) $${\left(X-x\right)}^{2}+{\left(Y-y\right)}^{2}=r^{2}$$

where *C* is a constant. From Equation (5), the position of the receiver can be obtained:

(6) $$\{\begin{array}{c} {\left(X_{A}-x\right)}^{2}+{\left(Y_{A}-y\right)}^{2}=r_{A}^{2}\\ {\left(X_{B}-x\right)}^{2}+{\left(Y_{B}-y\right)}^{2}=r_{B}^{2}\\ {\left(X_{C}-x\right)}^{2}+{\left(Y_{C}-y\right)}^{2}=r_{C}^{2} \end{array}$$

where $\left(X_{A},Y_{A}\right)$, $\left(X_{B},Y_{B}\right)$, and $\left(X_{C},Y_{C}\right)$ are the positions of LED A, LED B, and LED C; the terms $r_{A}$, $r_{B}$, and $r_{C}$ represent the distance from the LED A/B/C to the module; and (x,y) is the position of the module. To facilitate discussion, we regard the VLC position at time *k* as $Z{\left[x_{k},y_{k}\right]}^{T}$.

### 2.3. Inertial Navigation Algorithm

The classic pedestrian dead reckoning (PDR) method was reported in [11,12]. The PDR automates the self-localization based on the previous known position, the distance traveled and the direction of travel. In this work, a more popular foot-mounted PDR system is employed [13]. In the PDR system, a digital motion engine (DMP) of an MEMS module is used to obtain the real-time travel direction, and the distance traveled is obtained by a step detection and the stride estimation algorithm. Since the step frequency of an adult is approximately 1–3 steps per second, we employ a 5 Hz low pass filter to eliminate the high-frequency noise from the direction signal. The direction of travel at time *k* is denoted as $\theta_{k}$. A method based on an acceleration signal pattern has been proposed to detect step frequency in [14].

**Figure 2:**
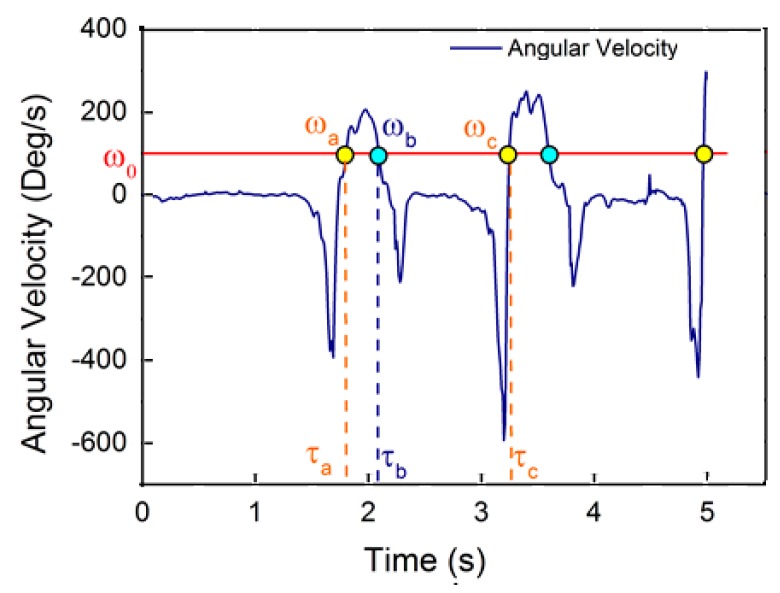
Step detection based on inertial navigation algorithm.

Since a cycle of human walking is composed of a standing phase and a walking phase, the walker’s foot will not move along a fixed direction or at a fixed speed during the entire walking period. The motion of foot in the walking period is cyclic [11], and the periodism will be reflected in the change in angular velocity and foot acceleration. In the actual tests, we found that the yaw angular velocity in the heading direction of the walker’s foot during walking is cyclic and has less high-frequency noise than the acceleration. Therefore, we count the step frequency using a method based on the threshold detection of the yaw angular velocity in the heading direction, as shown in Figure 2. We set an angular velocity threshold $\omega_{0}$ and a time interval threshold $\tau_{0}$. If the detected angular velocities $\omega_{a}$ and $\omega_{b}$, equal to $\omega_{0}$ , are at neighboring times $\tau_{a}$ and $\tau_{b}$, respectively, with $\tau_{b}-\tau_{a}<\tau_{0}$, the follow-up angular velocity detection will continue until the next time $\tau_{c}$, when the corresponding angular velocity satisfies $\omega_{c}=\omega_{0}$. If $\tau_{c}-\tau_{a}\geq \tau_{0}$ holds, a step will be counted. In Figure 2, the counted step points are colored in yellow, and the discarded point is colored in blue. There are two commonly used methods of stride estimation [15,16]; we use the Kim approach to estimate the stride $S_{k}$ for a general walker [11].

### 2.4. Fusion Position Algorithm Based on the Kalman Filter

As a common data fusing method, the Kalman filter is becoming the most commonly used method to fuse different positioning data of the diverse types of positioning systems or sensors [17,18,19]. In this section, the algorithms and models of the Kalman filter used in our fusing system are described. A simple process model with errors modeled as white noise is applied in the system. The state vector $X_{k}$ contains three elements: $x_{1k}$ is the heading angle, i.e., the foot’s orientation of the *k*-th time, $x_{2k}$ and $x_{3k}$ are the x-axis and y-axis coordinates in two-dimensional space. The filter is started from the initial estimate $X_{0}$ and the initial covariance $P_{0}$, which are set according to the best available estimate about the initial position and the uncertainty of the initial position information [14]. The state formula we used is that of Equation (7):

(7) $$X_{k}^{-}=X_{k-1}+\left[\begin{array}{c} \theta_{k}\\ S_{k}\cos x_{1k-1}\\ S_{k}\sin x_{1k-1} \end{array}\right]$$

where $X_{k-1}$ denotes the posterior estimate after the measurement update using the (*k* − 1)-th measurement samples, while $X_{k}^{-}$ is the prior estimate for the *k*-th time step, and $x_{1k-1}$ is the previous posterior estimate of the heading. The state matrix $F_{k}$ is obtained by taking the partial derivative of Equation (7), i.e., the Jacobian matrix, as follows:

(8) $$F_{k}=\left[\begin{array}{ccc} 1 & 0 & 0\\ -S_{k}\sin x_{1k}^{-} & 1 & 0\\ S_{k}\cos x_{1k}^{-} & 0 & 1 \end{array}\right]$$

As the effect of the step length uncertainty is multiplied by the sine and cosine functions of the heading, the state noise $Q_{k}$ is also approximated on every propagation step:

(9) $$Q_{k}=\left[\begin{array}{ccc} V_{\theta } & 0 & 0\\ 0 & \cos^{2}(x_{1k}^{-})V_{s} & 0\\ 0 & 0 & \sin^{2}(x_{1k}^{-})V_{s} \end{array}\right]$$

where $V_{\theta }$ is the variance of the heading angle measurement, and $V_{S}$ is the variance of the step length estimate. The covariance propagation to obtain the prior covariance $P_{k}^{-}$ is shown as Equation (10), as follows:

(10) $$P_{k}^{-}=F_{k}P_{k-1}F_{k}^{T}+Q_{k}$$

where $P_{k-1}$ is the posterior covariance from the previous time step. The measurement input of the filter is $Z={\left[x_{k},y_{k}\right]}^{T}$, which comes from the VLC positioning system. The measurement matrix is

(11) $$H=\left[\begin{array}{ccc} 0 & 1 & 0\\ 0 & 0 & 1 \end{array}\right]$$

**Figure 3:**
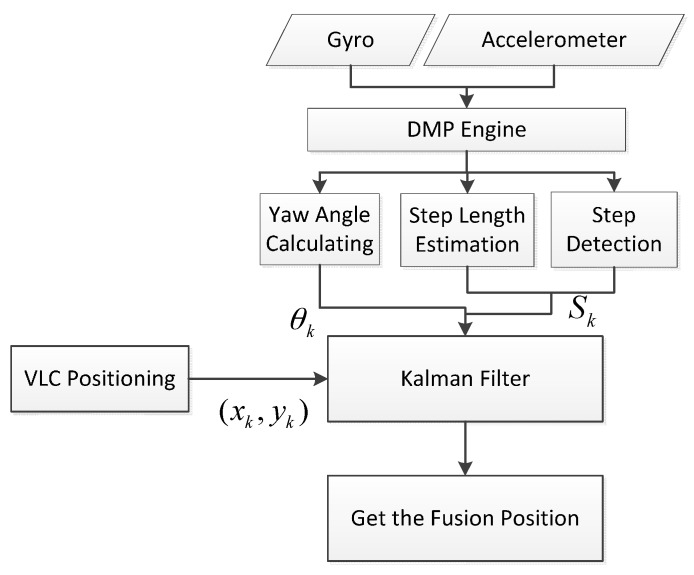
The algorithm flowchart of the fusion positioning.

The equations for the measurement update of the state $X_{k}$ and covariance $P_{k}$ are propagated by the Kalman filter formula as in Equation (12), which are as follows:

(12) $$\begin{array}{l} K_{k}=P_{k}^{-}H^{T}{(HP_{k}^{-}H^{T}+R)}^{-1}\\ X_{k}=X_{k}^{-}+K_{k}(Z_{k}-HX_{k}^{-})\\ P_{k}=(I_{3\times 3}-K_{k}H)P_{k}^{-} \end{array}$$

where R is the covariance of the VLC positioning coordinate estimates, $K_{k}$ is the Kalman gain matrix of the k-th time, and $I_{3\times 3}$ is the identity matrix. The algorithm flowchart of the fusion positioning algorithm is shown in Figure 3.

## 3. Field Experiment and Results

### 3.1. Experiment Setup

#### 3.1.1. Structure of the Fusion Positioning System

**Figure 4:**
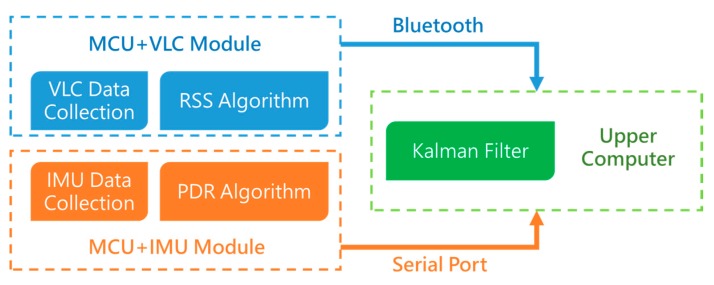
The system structure of the fusion positioning system.

Figure 4 shows the structure of the fusion positioning system we demonstrated in the experiment. Considering that the altitude of VLC positioning module will affect the positioning performance, the VLC-based positioning module and the inertial navigation module we designed are individual modules, respectively, the walker can wear the VLC positioning module on the hand, shoulder, or head, among others, so the walker can hold the attitude of VLC positioning module easily. The VLC-based positioning module and inertial navigation module each send respective localization data to a host computer. In order to provide a portable and wearable positioning system, we use Bluetooth to connect the VLC-based positioning module and the host computer in our experiment, and the inertial navigation module is connected to the host computer via serial port. In the host computer, the fusion positioning algorithm based on an extended Kalman filter iteratively runs to estimate the walker’s position.

**Figure 5:**
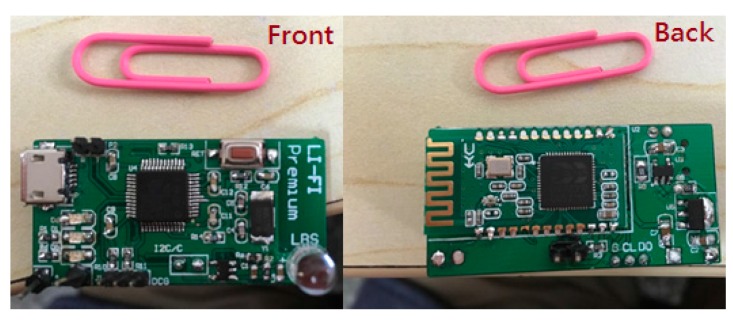
Visible light positioning module.

**Figure 6:**
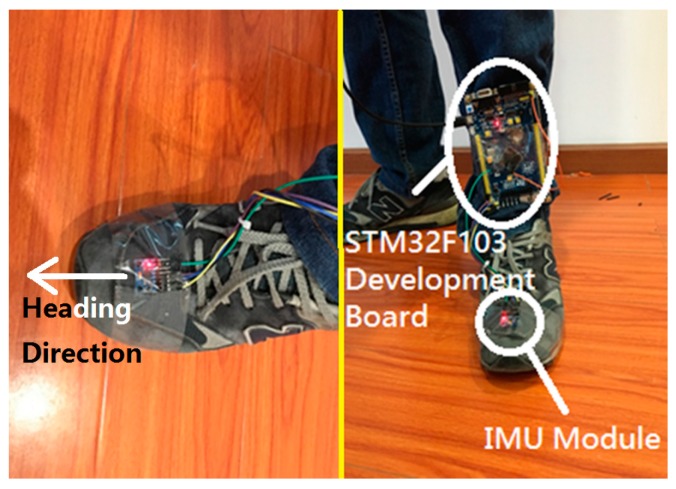
Inertial navigation module.

When the system starts to work, it will collect data first; the VLC positioning data will be collected and processed by the VLC module as shown in Figure 5. The PDR data will be collected by the MPU6050 MEMS module as shown in Figure 6, which are tied on the walker’s tiptoe. The module will collect the tiptoe’s inertial movement data and send the data to the STM32F103 MCU by the I2C bus. The MCU will calculate the positioning data to the host computer via serial bus.

The MPU6050 module we used contains Invensense’s Digital Motion Processor (DMP) engine. This engine will fuse the accelerated velocity data and angular velocity data and will send the posture data by an I2C or SPI bus.

#### 3.1.2. Experiment Environment

**Figure 7:**
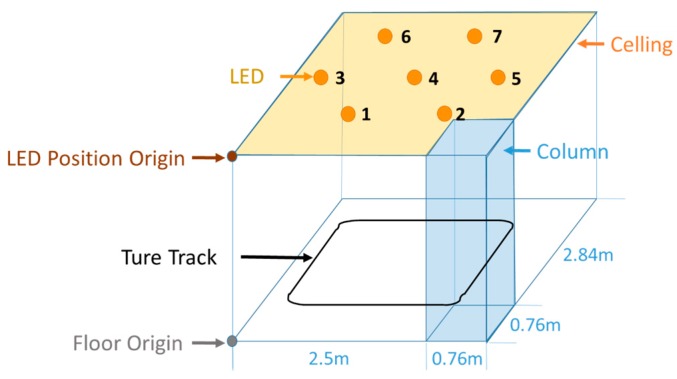
Experimental site layout model.

**Figure 8:**
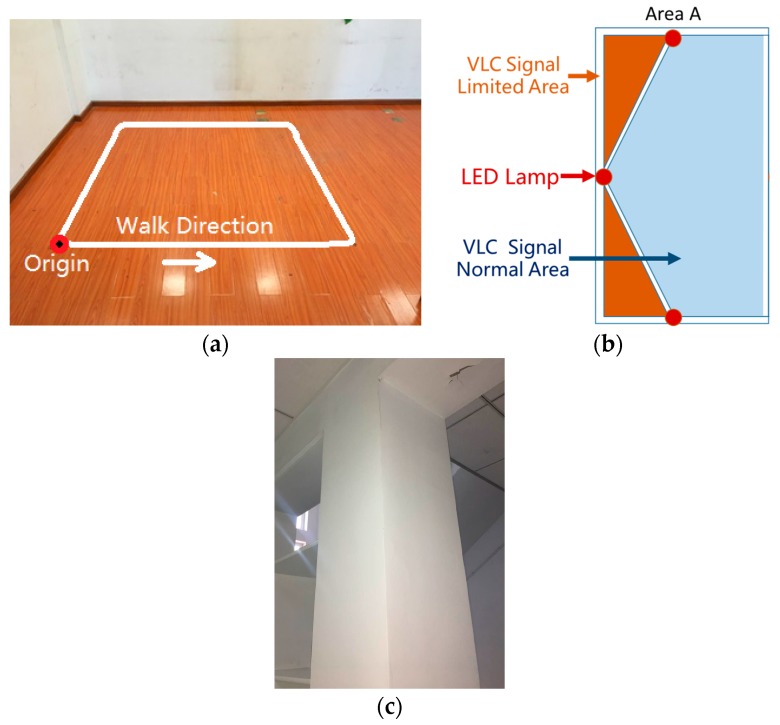
(**a**) Walking Path; (**b**) Diagram of the left of experiment field; (**c**) The column in the experiment field.

A test walk was conducted in the laboratory. As shown in Figure 7, the VLC positioning available area is a hexagon with a side length of 1.5 m, where there are two sides close to the wall and a corner close to a column. Seven LED down bulbs with a power of 17 W mounted on the ceiling serve as the lighting sources, and the ceiling height is 2.5 m. To set the origin at one corner of the room, the coordinates of seven LED sources in units of meter are (0.93, 0.888), (2.33, 0.888), (0.23, 2.1), (1.63, 2.1), (3.03, 2.1), (0.93, 3.312), and (2.33, 3.312). The walking route is a square with a 2.2 m side length as shown in Figure 8. In order to mimic the longer track, the tester walked two laps.

### 3.2. Experiment Results

**Figure 9:**
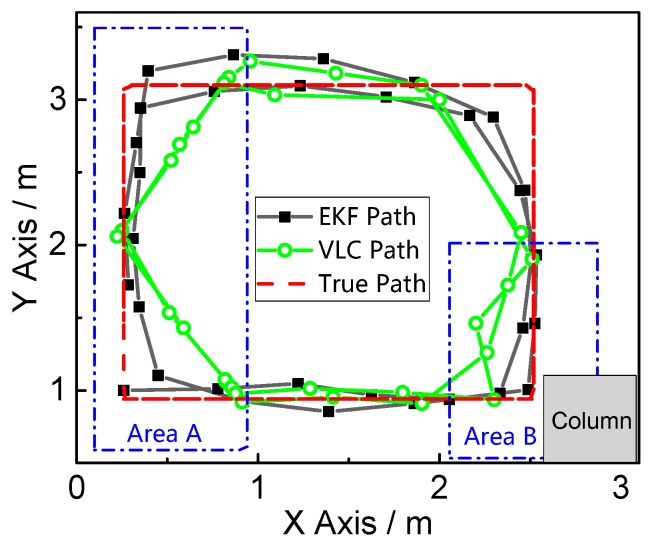
Fusion positioning (EKF Path) and VLC positioning (VLC Path) result.

The final positioning results from the fusion positioning method and VLC is shown in Figure 9.

**Figure 10:**
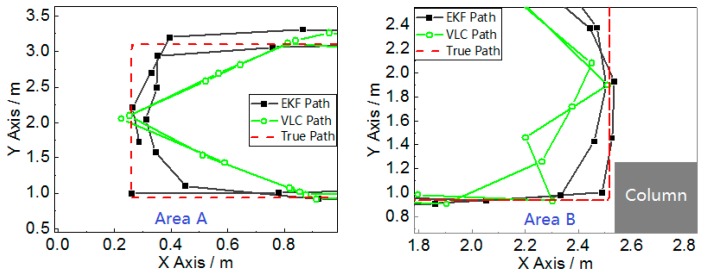
Magnification of Area A and Area B in Figure 9.

The experiment we conducted can verify the two problems of the classical VLC positioning mentioned in Section 1: the left of this field is named “Area A”, and there are two VLC signal-limited areas, shown as the orange areas in Figure 8b, which are outside of the hexagon area made by the seven LEDs. The VLC positioning module can only receive two LED bulbs’ light signals effectively in the VLC signal limited area, which is why Area A can verify the system’s function in the LED projected hexagon area. The right bottom of this field, named “Area B”, is close to the column shown in Figure 8c. The reflection of the column easily impacts the distribution of a visible light signal. This area can verify the system’s anti-multipath effect function. Figure 10 magnifies Area A and Area B in Figure 9, which indicates that the fusion position system yields superior positioning performance in both areas.

**Figure 11:**
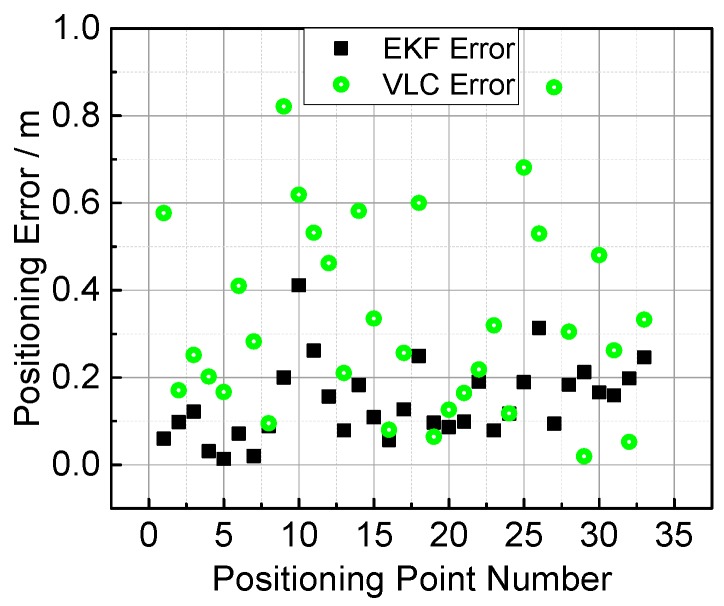
Measured error with fusion positioning (EKF Error) and VLC positioning (VLC Error) methods.

**Figure 12:**
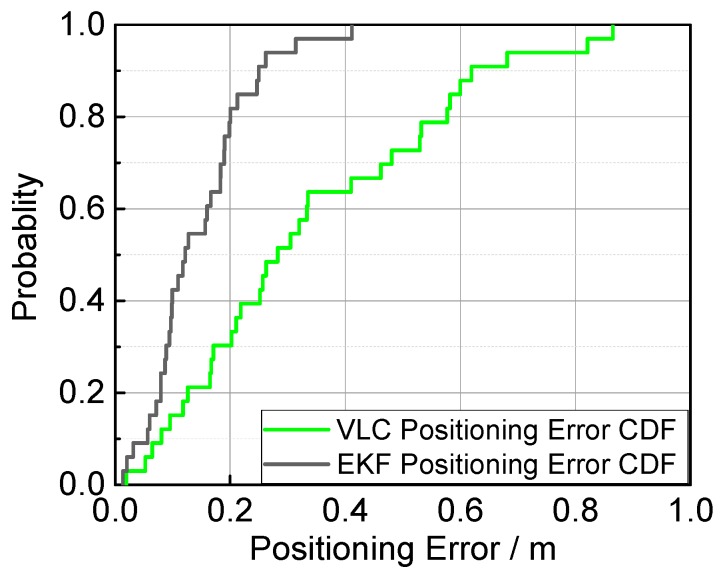
Error cumulative distribution functions of fusion positioning and VLC positioning methods.

For a better comparison, the positioning error and the error cumulative distribution functions (CDFs) are plotted in Figure 11 and Figure 12. The error properties are shown in Table 1. Regarding the mean error, the fusion positioning provides an improvement of 57.3% relative to VLC positioning. In Area B, the maximum positioning errors are 0.0849 m and 0.2218 m, decreased by more than 60% with hybrid positioning. In Area A, the positioning result of VLC positioning excludes the area outside the hexagon layout area, and the fusion positioning can provide an accurate position even outside the hexagon projection area with the aid of inertial navigation.

**Figure 13:**
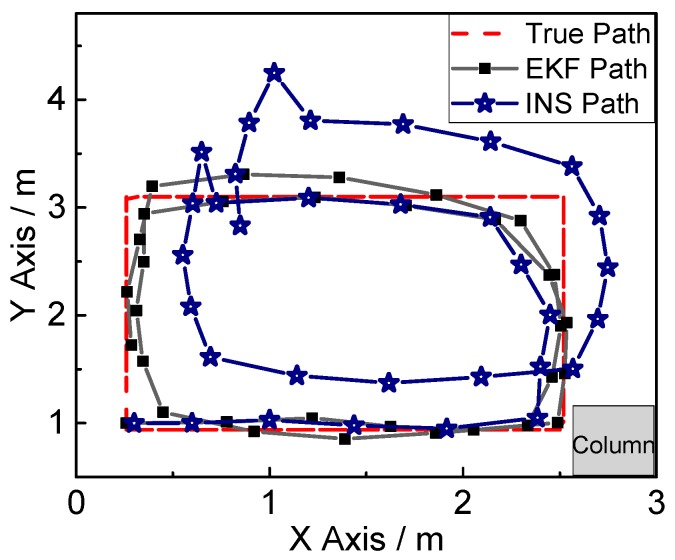
Fusion positioning and inertial navigation result.

Last but not the least, the positioning result of inertial navigation is shown in Figure 13. The positioning accuracy of inertial navigation is high at the first 3/8 lap, but the positioning error increases in the following route. It is observed that the positioning result after the first 3/4 lap greatly deviates from the true track. 

The above experimental results indicate that, with the fusion positioning scheme based on the extended Kalman filter, the problems of VLC positioning and inertial navigation can be solved. Both the positioning accuracy and stability are improved with the comparison of VLC positioning and inertial navigation. In the experiment, the measurement is carried out for a general walker with common walking behavior, so the positioning performance should remain the same if the fusion positioning system is applied in practical scenarios with a larger area.

**Figure 14:**
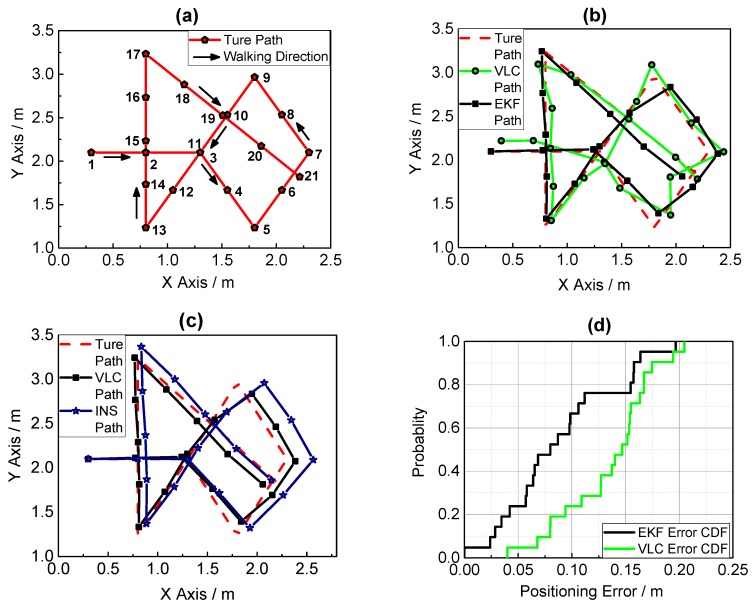
(**a**) The true path of walking; (**b**) Fusion positioning and VLC positioning result in a complex trajectory; (**c**) Fusion positioning and PDR positioning result in a complex trajectory; (**d**) Error cumulative distribution functions of VLC and PDR positioning.

In order to verify the positioning performance in complex and extreme condition, we also conducted another experiment with a more complicated path, and the entire path was inside the hexagon area made by the seven LEDs, which was designed with little VLC signal limited area to simulate true application scenarios. As Figure 14 shows, the experiment result shows that the fusion positioning provides an improvement of 34.58% relative to VLC positioning regarding the mean error, which indicates that the fusion positioning system can also provide a better positioning result in a complex trajectory and condition.

## 4. Conclusions

In this paper, we propose a method and system fusing the VLC-based indoor positioning and inertial navigation with the deployment of an extended Kalman filter. The experiment on a general walk demonstrate that the fusion positioning system can decrease the error caused by a multipath effect, and this system can also position paths normally outside of the layout of the LEDs, the accuracy of hybrid localization system remains the same in practical scenarios of larger area.

In summary, this system can circumvent the problems of the classic VLC positioning and improve the reliability and applicability of VLC positioning, which helps the marketability and development of VLC positioning. The fusing positioning system we proposed can be used in consumer-grade electronics, such as client guides in markets, museums, and indoor navigation systems in hospital, and it can also be used for industrial purposes, i.e., robots or AGV self-navigation.

We are working to improve the robustness and accuracy of the fusion positioning system. The new fusing algorithm such as unscented Kalman filter and neutral network is going to be applied to the positioning method. In addition, the performance of VLC positioning and inertial navigation themselves is also of interest. For visible light positioning, we are trying to increase the number of iterations. For inertial navigation, we can choose high-accuracy IMU modules in different practical applications and develop other optimization algorithms such as particle filter.

## Figures and Tables

**Table 1:** Positioning error comparison.

Error	VLC	EKF
Maximum	0.619 m	0.411 m
Average	0.339 m	0.145 m
Minimum	0.167 m	0.137 m
